# Classification of *Suncus murinus* species complex (Soricidae: Crocidurinae) in Peninsular Malaysia using image analysis and machine learning approaches

**DOI:** 10.1186/s12859-016-1362-5

**Published:** 2016-12-22

**Authors:** Arpah Abu, Lee Kien Leow, Rosli Ramli, Hasmahzaiti Omar

**Affiliations:** Institute of Biological Sciences, Faculty of Science, University of Malaya, 50603 Kuala Lumpur, Malaysia

## Abstract

**Background:**

Taxonomists frequently identify specimen from various populations based on the morphological characteristics and molecular data. This study looks into another invasive process in identification of house shrew (*Suncus murinus*) using image analysis and machine learning approaches. Thus, an automated identification system is developed to assist and simplify this task. In this study, seven descriptors namely area, convex area, major axis length, minor axis length, perimeter, equivalent diameter and extent which are based on the shape are used as features to represent digital image of skull that consists of dorsal, lateral and jaw views for each specimen. An Artificial Neural Network (ANN) is used as classifier to classify the skulls of *S. murinus* based on region (northern and southern populations of Peninsular Malaysia) and sex (adult male and female). Thus, specimen classification using Training data set and identification using Testing data set were performed through two stages of ANNs.

**Results:**

At present, the classifier used has achieved an accuracy of 100% based on skulls’ views. Classification and identification to regions and sexes have also attained 72.5%, 87.5% and 80.0% of accuracy for dorsal, lateral, and jaw views, respectively. This results show that the shape characteristic features used are substantial because they can differentiate the specimens based on regions and sexes up to the accuracy of 80% and above. Finally, an application was developed and can be used for the scientific community.

**Conclusions:**

This automated system demonstrates the practicability of using computer-assisted systems in providing interesting alternative approach for quick and easy identification of unknown species.

## Background

As stated by [1], images are central to a wide variety of fields ranging from history [2, 3] to medicine [4, 5], including astronomy [6, 7], oil exploration [8, 9] and weather forecasting [10, 11]. Image plays an important role in numerous human activities such as law enforcement [12, 13], agriculture and forestry management [14, 15], earth science [16] and so forth. One of the uses of images is in face recognition and identification [17, 18].

Similarly, in biology, images are needed for educational and scientific research purposes. In biodiversity research, vast numbers of images produced by scientists provide useful information to many contemporaries. From the (biological) images, elements such as diagnostic hard parts can be used for organism identification at certain level such as genus or species. The findings contribute to scientific research as well as teaching and educational purposes. With the advancement in computer vision [19], image processing [20] and machine vision studies which involve studies such as artificial intelligence, imaging and pattern recognition, digital images in biology can be applied for species identification which is needed to assist and support biologists in doing their research.

There are many research groups working on species identification for both plan7s and animals. Briefly, automated identifications have been developed for the identification of plants (based on shapes, texture and colours of leaves) [21–24], helminth parasites (based on eggs shape and texture) [25], butterfly families (based on colour, texture and shape of wings) [26] and marine life based on colours of the images [27].

One of the established species identification systems is DAISY. DAISY is widely used for species identification [28]. It can be used to help non-experts for rapidly screening the unknown species. The prototype was first developed and tested to discriminate five species of parasitic wasp, based on differences in their wing structure using principle component analysis and linear discriminant analysis [29]. DAISY was also used in the identification of other insect groups such as the biting midges, Xylophanes hawkmoth [30] and live moths of Macrolepidoptera [31, 32]. DAISY system is generic [33] and further enhanced with new methods such as artificial neural network and support vector machines [34], and plastic self-organizing map [23]. As mentioned by [28], DAISY has been exhaustively tested in many significant morphological and molecular datasets including British bumblebees [35], British Lepidoptera (butterflies), sphingid larvae and lycosid spiders.

Another example for generic species identification is SPIDA [36]. SPIDA, which is an identification system for spiders whereby artificial neural network is applied to recognize images, encoded with wavelet [37]. Until 2005, they have developed internet-accessible automated identification system named SPIDA-web (Species IDentification, Automated and web accessible) with two perspectives i.e. taxonomic (Family Trochanteriidae) and geographic (surveys conducted in Knox Co., TN).

ABIS is an identification system of bee species by image analysis of their wings. This system is also integrated and applied as a tool for data gathering within the information system EDIS - Entomological Data Information System. Geometrical image analysis, template matching, affine projection, discriminant analysis, kernel functions and GIS are the methods used in developing this system [38].

The last example is DrawWing, which is the software for insect identification based on the analysis of wing images and currently it is working on honeybee (*Apis*) wings [39]. Generally, with advancement in information technology, many systems and tools have been developed to assist and support biologists in performing their research works.

Both DAISY and SPIDA are generic-based system, which means these systems can be used to recognise many other species. On the contrary, ABIS and DrawWing are restricted to insects, which operate by matching specific set of characteristics based on wing venation. Basically, the identification system is built based on pattern recognition approach. The species diagnostic characters are used for the identification, which are represented by certain patterns such as colour, shape and/or texture. The query image will be compared to the images in the training set and the identification result; normally the system will return the identified species image along with taxon species name but no complete annotations to describe the image.

There are a few aspects that are important to consider when developing an automatic identification system, i.e. the images for both training and testing data sets, features to represent the image, the classifier, query specification and the expected output of the identification process. The images are the main input requirement, whereby all images must be with the same standard properties. Therefore, image pre-processing is needed to ensure the width, height and pixel size of all images are the same standards. The images should also free from any noise. With regards to pattern recognition, features are needed to represent an image, the similarity between two images are then compared using distance function and the similar images to the query image are classified using classifier. As for query specification, a query image is needed as input whether in query-by-example, query-by-sketch or query-based browsing method. The last aspect is the output of the identification process, which is crucial in determining whether the identification process works well and in an efficient manner. Thus to achieve this, the most similar and relevant species must be identified.

From the above study, some pertinent research questions asked are:-

- How unique features could be extracted from a two-dimensional digital image? It is necessary to decide whether to use boundary-based information or region-based information to determine the shape characteristics for classifying and comparative purposes.
- How feature selection techniques and classifiers could be applied to perform classification and identification? The accuracy of the classification is depending on the numbers of features and the classifier to classify the skull of *S. murinus* images.

The present study has the objectives of (1) extracting shape characteristics (morphological information) from the dorsal, lateral and jaw views of skull by using image processing techniques in order to perform an automatic *S. murinus* species identification based on different populations of Peninsular Malaysia, and (2) to examine the variations in the combination of shape of the dorsal, lateral and jaw views and sexes of male and female among the different regions of its distribution.

## Methods

### An overview of the classification based on shape characteristics

**Fig. 1:**
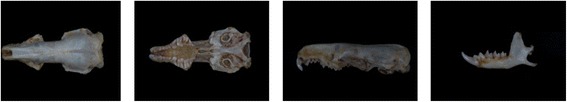
Digital image of *S. murinus* skull. Skull in four views namely dorsal, ventral, jaw and lateral

Figure 1 presents digital images of *S. murinus* skull. The views can be made in four different angles; i.e. dorsal, ventral, jaw, and lateral, depending on the shape of the specimen. However, ventral view was excluded in this study as the use of only dorsal view assumes that both dorsal and ventral views have the identical shape.

**Fig. 2:**
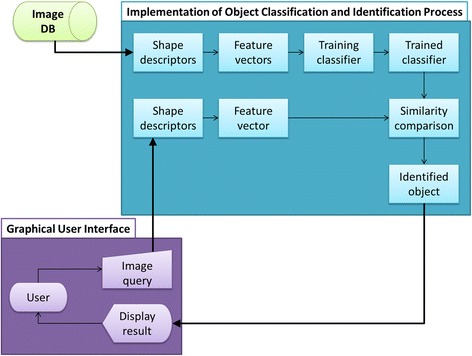
Procedural flow of the study. It involved the development of image database, the implementation of the object classification and identification process and development of graphical user interface

In order to identify the specimen that corresponds to a specific view, four networks were created to characterise each view shape, and then applied it for specimen classification. The implementation of the object classification and identification process is shown in Fig. 2. This includes image segmentation, image analysis, training of classifier, object classification and identification. A graphical user interface was also developed for system testing and evaluation. Matlab R2015b has been used to develop and implement the techniques and methodologies mentioned above as it provides various built-in algorithms for image processing and computer vision application [40].

### Shape characterization

#### Sampling sites and specimen collection

**Fig. 3:**
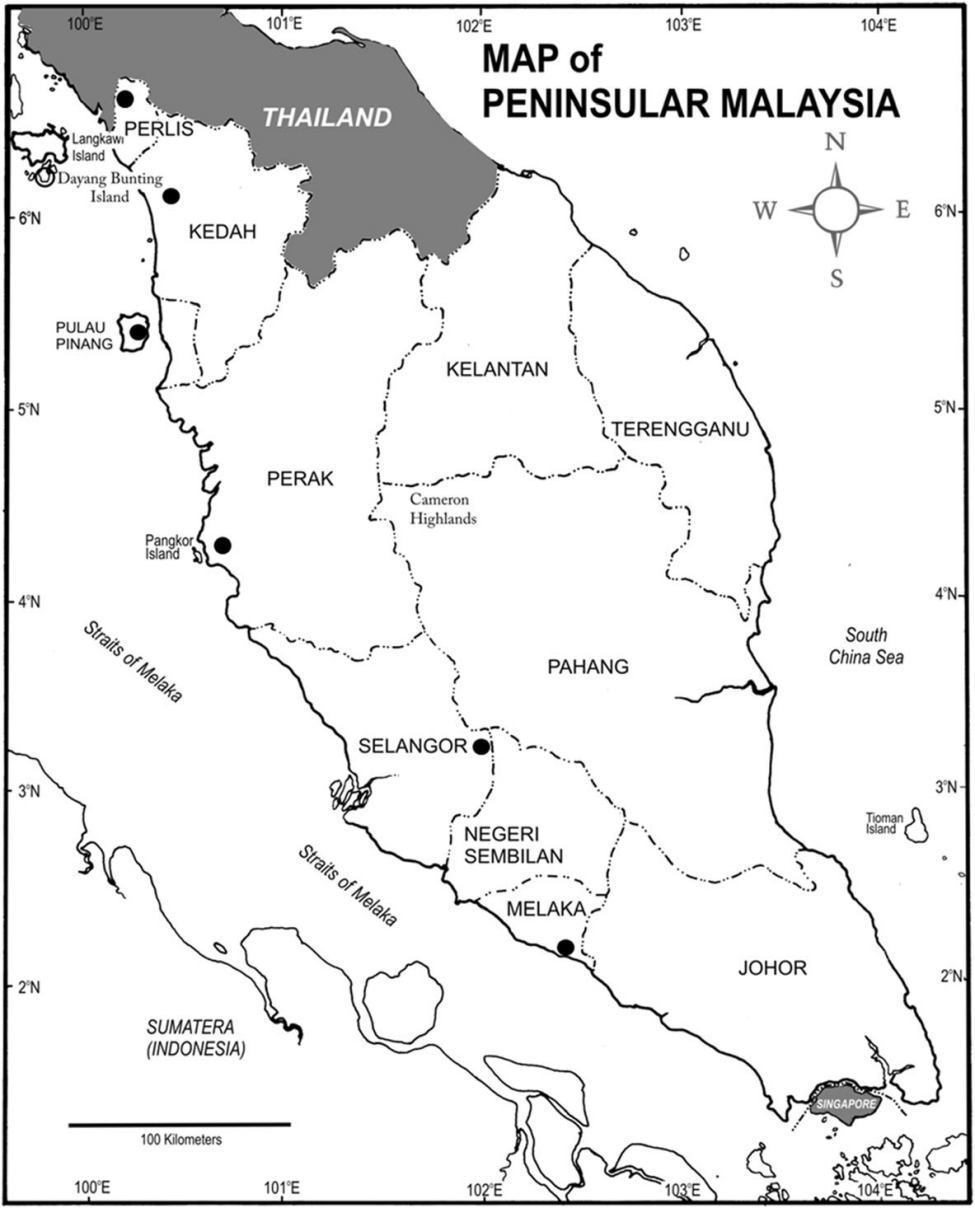
Location of the sampling areas in Peninsular Malaysia. Wang Kelian, Perlis, Alor Setar, Kedah and Air Hitam, Pulau Pinang are grouped into Northern region; and Lumut, Perak, Ulu Gombak, Selangor and Bukit Katil, Melaka are grouped into Southern region

A total of 100 specimens of house shrew were collected from six locations in the west coast of Peninsular Malaysia (Fig. 3). These locations were divided into three different habitats i.e., (1) urban, located in rapidly urbanizing area; (2) suburban, surrounded by rural area; and (3) edge of secondary forest (see Table 1). At each sampling site, 60 cage traps (with dimensions of 30 x 15 x 15 cm) were placed on the ground for four to five successive nights across human settlements such as restaurants, stool plant area, landfills, water drain hose and garden near to trench. All cages were baited with fried chicken or fried fish to attract the shrews to enter the traps.

**Table 1:** Sites where *S. murinus* were collected throughout west coast of Peninsular Malaysia and respective number of specimens

Location	Habitat Types	No. of Specimens	
		Female	Male
*Northern region*			
Wang Kelian, Perlis	Edge of forest	12	4
Alor Setar, Kedah	Suburban	3	12
Air Hitam, Pulau Pinang	Urban	5	14
*Southern region*			
Lumut, Perak	Suburban	10	16
Ulu Gombak, Selangor	Edge of forest	3	5
Bukit Katil, Melaka	Suburban	9	7

A total of 100 specimens of house shrew were collected from six locations in the west coast of Peninsular Malaysia. These locations were divided into three different habitats i.e., urban (located in rapidly urbanizing area); suburban (surrounded by rural area); edge of secondary forest

### Dissecting, skull extraction and museum collection

Photo for alive shrew was taken in modified aquarium before shrew was euthanized using zoletil. After being euthanized, all information of freshly dead shrew was recorded immediately based on external morphology such as head and body length, hind foot length, fore foot length, tail length, ear length, body weight, fur colours (e.g. dorsal and ventral part, hind limb and fore limb and tail) and the presence of hair at upper tail. Specimens were then prepared as a flat skin and for the residues were preserved in 70% ethanol. Meanwhile, all extracted skulls from each specimen were placed into small bottles separately for imaging analysis. The specimens of house shrew include dried, wet and skull are currently kept at the Museum Zoology, University of Malaya, Kuala Lumpur. Table 1 shows the number of specimens that were collected from six different locations, which were grouped into northern and southern region populations.

### Image acquisition

Skull digital images were used as the starting point for the automatic analysis. All images were taken by the same person to avoid any variation associated with possible different points of view of observers. Specimen placement was standardised, whereby the dorsal and ventral of skull were captured on the right side, from the distance of 35 cm from the camera lens and oriented at an angle of 90°, and the lateral and jaw of skull were captured on the right view, from the distance of 27 cm from the camera lens and oriented at an angle of 0°. The images were captured using Nikon D90 with a resolution of 4288 x 2848 pixels, scaled to a spatial resolution of 240 pixels/inch and stored as 32-bit RGB colour Tagged Image File Format (tiff) format. Due to illumination and contrast problem, which would hamper the process of object segmentation, Adobe Photoshop CS6 was used to enhance the image quality. Consistency of image quality is important to ensure the accuracy of the classification and identification process.

Once the enhanced image quality was verified by shrew expert, the processed images were stored in file folder in the hard drive. These images were indexed according to their views, regions and sexes. 60% of the specimens for each region and sex were used as Training data set and another 40% were used as Testing data set.

### Region-based features

The image processing and analysis steps involved in this study were divided into two main components which are described as follows.

#### Image segmentation

For each of the images from both Training and Testing data sets, a series of image pre-processing steps were carried out automatically. First, an image in spatial domain was converted from RGB to grayscale using the *rgb2gray* function. To detect the object in the image, a gradient based segmentation *ordfilt2* function was used whereby *2-D order-statistic* filter with *10-by-10 domain* was applied. The edge of the image was obtained from the difference between the first and the last order filter. The image with detected edge was then converted to a binary image using the *im2bw* function. From the binary image, the object (shape of the view) was obtained after filling the holes in the image and clearing the border using the *imfill* and *imclearborder* functions. To ensure that the desired view shape was the only object in the image, small particles in the surrounding were removed using the *bwareaopen* function before features extraction was carried out. Through this image segmentation process, the object or the region of interest (ROI) was obtained.

#### Features extraction

Features were measured from the ROI – shape of the view using region properties function named *regionprops*. The extracted features are area, convex area, major axis length, minor axis length, perimeter, equivalent diameter and extent. For each of the images in the Training data set, the extracted feature values were arranged and saved in MS Excel spreadsheet and then were used for neural network training. Whereas for images in the Testing data set, the extracted feature values were used to simulate the trained network to get the output values of the network which represented the region and sex of the input images. Results from the network simulation were compiled in MS Excel spreadsheet and then was compared with the result from the experts and the accuracy of the identification using the trained network can be determined.

### Classification using Artificial Neural Network (ANN)

ANN was chosen as the pattern recognition tool to train networks with the images from Training data set. Neural network training was done in two parts; first, the classification of images into three different views – Dorsal [D], Jaw [J] and Lateral [L], and, the second part is to classify the images into different regions and sexes – Northern Male [NM], Northern Female [NF], Southern Male [SM] and Southern Female [SF].

**Fig. 4:**
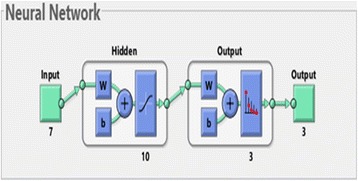
Neural network for all views (Three classes – D, L, J)

For the first part of the neural network training, there were 30 images for each specimen views of the Training data set. The developing network is a two-layer feed-forward network with seven input nodes (the extracted feature values) and three output nodes (desired classes – D, J, L) as shown in Fig. 4.

**Fig. 5:**
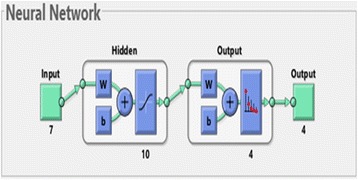
Neural network for views based on different regions and sexes (Four classes – NM, NF, SM, SF)

For second part of the neural network training, three different networks were trained based on the possible views of the specimen images. For each of the views, 30 images for each class were used in the training set. The networks were two-layer feed-forward with seven input nodes (the extracted feature values) and four output nodes (desired classes – NM, NF, SM, SF) as shown in Fig. 5. Before starting the neural network training, the images from the Training data set were divided into three sets; the training set, testing set and validation set with the percentage of 70%, 15% and 15%, respectively (the best percentage). The networks were trained for several times to obtain network with the best performance for each class. The performances of the trained networks were evaluated using Cross-Entropy and confusion matrices which showed the percentage of correct classification. The best trained networks for each class were saved and then used to simulate the extracted feature values from the images in Testing data set. The accuracy of identification was determined by the corrected classification by the trained network.

## Results

### Feature space and selection

**Fig. 6:**
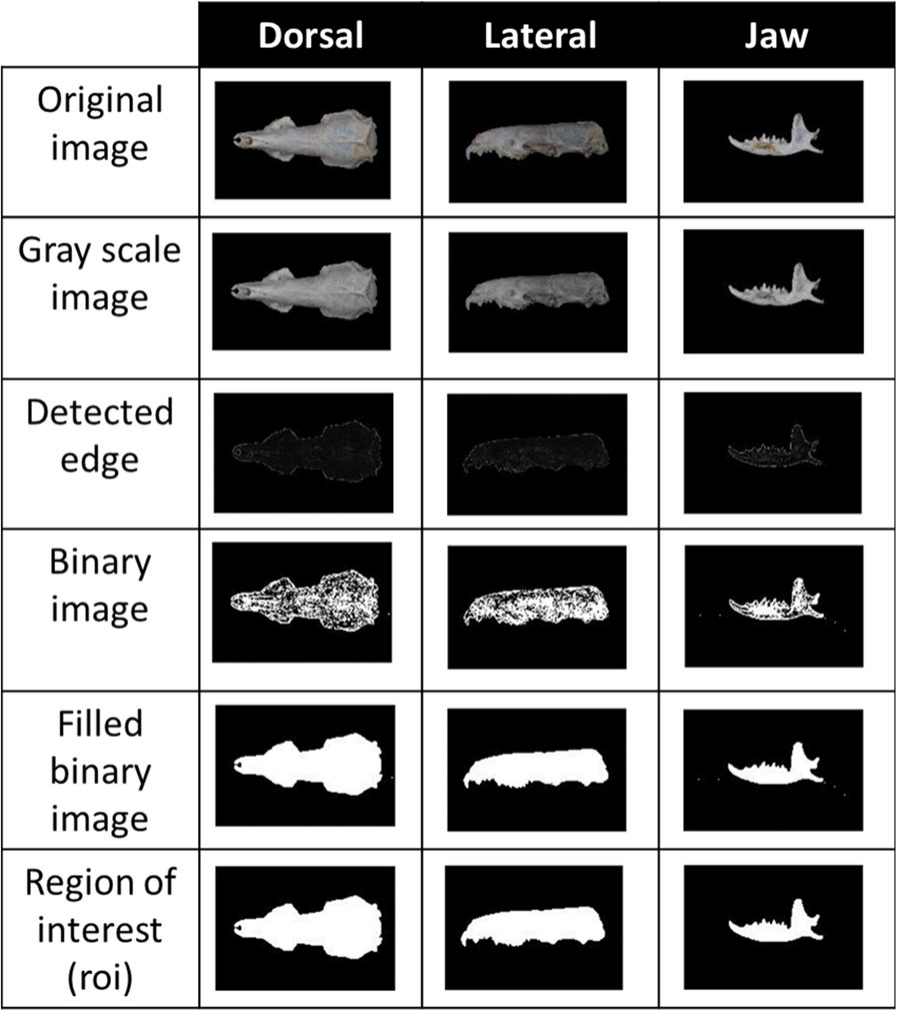
Steps in image segmentation. The ROI is obtained through image pre-processing, edge detection, conversion into binary image and object enhancement processes

The feature space is defined by all features namely area, convex area, major axis length, minor axis length, perimeter, equivalent diameter, and extent. After going through image segmentation step, the ROI in each images were obtained. The outcomes for each of the steps are visualized in the Fig. 6 below.

Features were then extracted from the ROI of the image using region properties function in Matlab which allows users to extract more than twenty shape measurement values from a single image. However, not all shape measurements were used as some measurements might not show difference amongst specimen images. Seven features which mainly size based were chosen and extracted for neural network training.

### Evaluation of the size of the training set used in ANN

For each of the neural network trainings, 60% of randomly selected specimen images were used as Training data set and 40% of randomly selected specimen images were used as Testing data set. All specimen images in Training data set were then used for performing evaluation of the size of the training set used in ANN. Thus, a series of tests with various percentages were conducted to estimate the minimum number of specimens required but still able generates an acceptable rate of correct classification as shown in Table 2. At first, 50% of the specimen images were randomly chosen and allocated as the training set. Additional specimen images were then shifted from testing and validation sets to the existing training set to make up the training set of 60%, 70%, 80% and 90%. This process was implemented recursively and classification performances of different percentages of training set were recorded. The result shows that allocating 70% of the specimen images as training set and 30% of the specimen images as the testing and validation sets resulted in the best performance for the classification and hence this combination was used in all the neural network training in this study.

**Table 2:** The effect of size of training set on the classification of shrews

Training	Testing	Validation	All views	Dorsal	Lateral	Jaw	Average
Numbers in percentage %							
50	25	25	*100*	*70.0*	*76.7*	*61.7*	77.1
60	20	20	*100*	*62.5*	*82.5*	*68.3*	78.3
*70*	*15*	*15*	*100*	*72.5*	*88.3*	*85.0*	*86.5*
80	10	10	*100*	*70.8*	*88.3*	*76.7*	84.0
90	5	5	*100*	*65.8*	*85.8*	*75.0*	81.7

A series of tests was conducted to estimate an acceptable rate of correct classification for Training data set

In this study, the performances of the networks were shown in the Cross-Entropy graph in which the best validation performance were obtained at the epoch where Cross-Entropy values for validation sets started to increase and the network generalisation stopped improving. The results of training performance and confusion matrix showing the percentage of correct classification in the training, testing and validation are explained as follows.

**Fig. 7:**
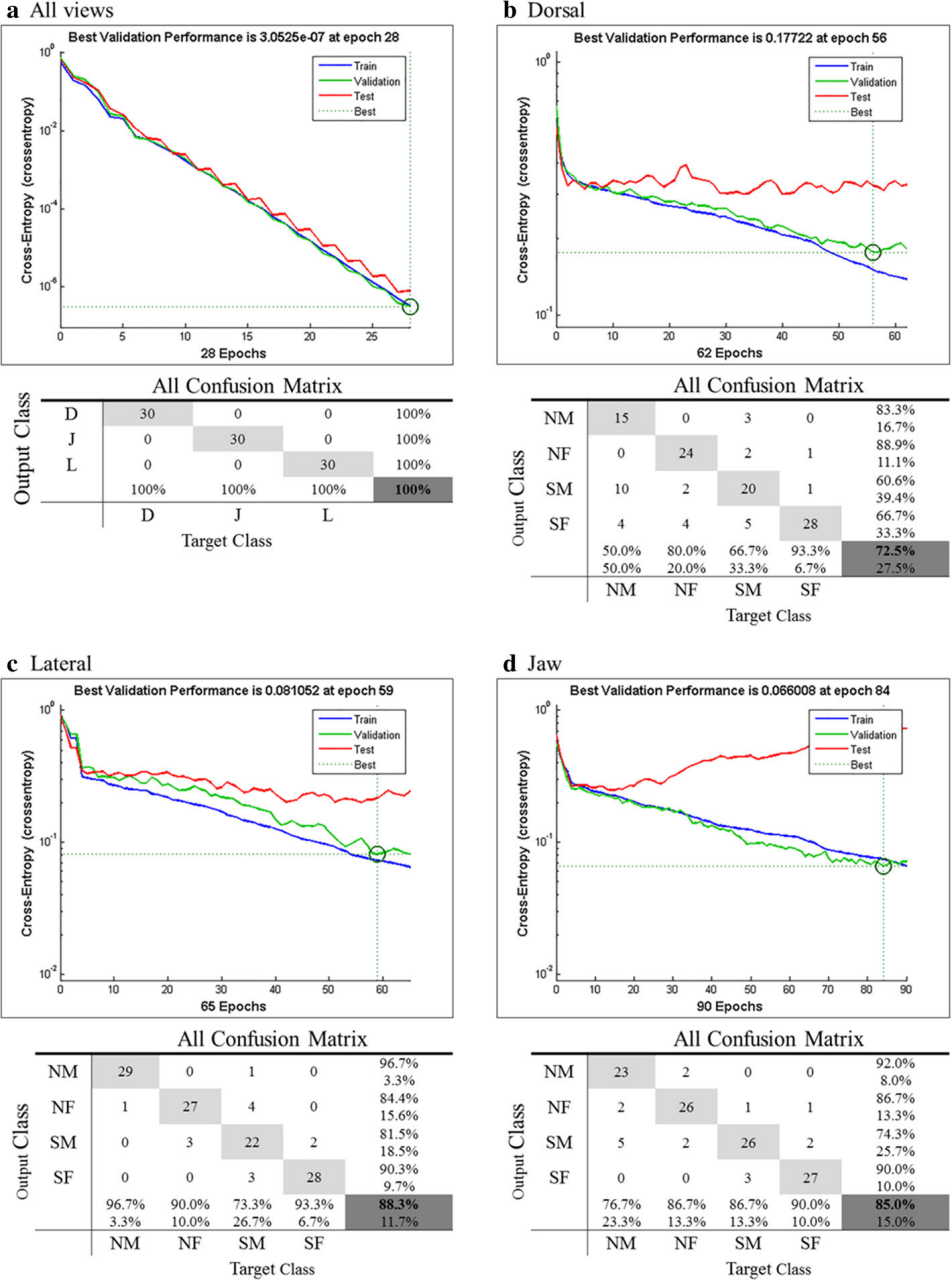
Training performance graphs and confusion matrix for all four networks

Classification of images into three different views (Dorsal, Jaw and Lateral) – the best trained network was obtained with 28 iterations and the best validation performance achieved when the cross-entropy value is 3.0525e-^07^ at epoch 28. A result of 100% correct classification in the training, validation and testing sets of all 90 images of D, J and L for both male and female from northern and southern regions as shown in Fig. 7(a).

Classification of dorsal images in different regions and sexes – the best trained network was obtained with 62 iterations and the best validation performance achieved when the cross-entropy value is 0.17722 at epoch 56. Result of confusion matrix shows that the overall rate of correct classification is 72.5% for all 120 dorsal images of NM, NF, SM and SF as shown in Fig. 7(b).

Classification of lateral images in different regions and sexes – the best trained network was obtained with 65 iterations and the best validation performance achieved when the cross-entropy value is 0.081052 at epoch 59. Result of confusion matrix shows that the overall rate of correct classification for all 120 lateral images of NM, NF, SM and SF is 88.3% as shown in Fig. 7(c).

Classification of jaw images in different regions and sexes – the best trained network was obtained with 90 iterations and the best validation performance achieved when the cross-entropy value is 0.066008 at epoch 84. Result of confusion matrix shows that the overall rate of correct classification for all 120 jaw images of NM, NF, SM and SF is 85% as shown in Fig. 7(d).

### Analysis of populations differentiation using Testing data set

Population differentiation was carried out to classify the specimens based on regions and sexes. This was done by using shape characteristics that were extracted from the digital images of dorsal, lateral and jaw. Species population differentiation tests were performed with Testing data set, comprising of 60 images for all views and 80 images of dorsal, lateral and jaw views. All these Testing data sets were randomly chosen and each one was used as an input for the identification process, which in turn generated a confusion matrix as a result of species discrimination. Thus, at the end of this recursive process, more than 200 confusion matrices were generated which were used to compute the average confusion matrix. This latter matrix contained the average of correct identification for all tested view images. As a final point, overall percentage of identification accuracy was obtained by computing the diagonal average.

This is to further prove the indication that same species of shrew in peninsular Malaysia shows population differentiation in terms of their morphological aspects. Confusion matrices visualise clearly on the misidentification percentage and the tendency of certain classes to be misidentified as another classes and further show the relationship between different classes; for example, specimens are prone to misidentification to the opposite sex from the same region.

Classification of images into three different views (Dorsal, Jaw and Lateral) – the trained network was tested with 20 specimen images for each view. The result in Table 3 shows the approach used in this study was capable of identifying the views correctly with 100% accuracy.

**Table 3:** Confusion matrix of specimen differentiation using all views Testing data set

Class	Result			Accuracy %
	Dorsal	Jaw	Lateral	
Dorsal	*20*	0	0	100
Jaw	0	*20*	0	100
Lateral	0	0	*20*	100
Overall				*100*

The approach used in this study was capable of identifying the views correctly with 100% accuracy

Classification of dorsal images in different regions and sexes – the trained network was tested with 80 images (20 specimen images for each NM, NF, SM and SF class). The result in Table 4 shows the overall accuracy is only 72.5%. Specimens NF, SM and SF were identified with 80%, 70% and 95% accuracy, respectively. It shows that there are substantial differences in terms of their morphological aspects. However, specimen NM shows poor result with only 45% accuracy and misidentified as SM and SF.

**Table 4:** Confusion matrix of specimen differentiation using dorsal Testing data set

Class	Result				Accuracy %
	NMD	NFD	SMD	SFD	
NMD	*9*	0	7	4	45
NFD	0	*16*	2	2	80
SMD	1	2	*14*	3	70
SFD	0	0	1	*19*	95
Overall					*72.5*

The overall accuracy is only 72.5% for specimen differentiation using dorsal Testing data set

Classification of lateral images in different regions and sexes – the trained network was tested with 80 images (20 specimen images for each NM, NF, SM and SF class). The result in Table 5 illustrates the best overall accuracy is 87.5%, compared to the other views. Among the southern specimens, SFs were identified with 100% accuracy which means that there is a substantial morphological difference between the male and female. As for the specimens from northern region, there are four specimens were misidentified as SM.

**Table 5:** Confusion matrix of specimen differentiation using lateral Testing data set

Class	Result				Accuracy %
	NML	NFL	SML	SFL	
NML	*19*	0	1	0	95
NFL	0	*17*	3	0	85
SML	1	3	*14*	2	70
SFL	0	0	0	*20*	100
Overall					*87.5*

The overall accuracy is 87.5% for specimen differentiation using lateral Testing data set. This is the best overall accuracy compared to the other views

Classification of jaw images in different regions and sexes – the trained network was tested with 80 images (20 specimen images for each NM, NF, SM and SF class). The result in Table 6 illustrates that the specimens were identified with overall 80% accuracy. It shows that there are substantial morphological differences for specimens from northern and southern regions including their sexes.

**Table 6:** Confusion matrix of specimen differentiation using jaw Testing data set

Class	Result				Accuracy %
	NMJ	NFJ	SMJ	SFJ	
NMJ	*15*	4	1	0	75
NFJ	2	*16*	2	0	80
SMJ	0	1	*16*	3	80
SFJ	0	1	2	*17*	85
Overall					*80*

The overall accuracy is only 80.0% for specimen differentiation using jaw Testing data set

### Identification system

**Fig. 8:**
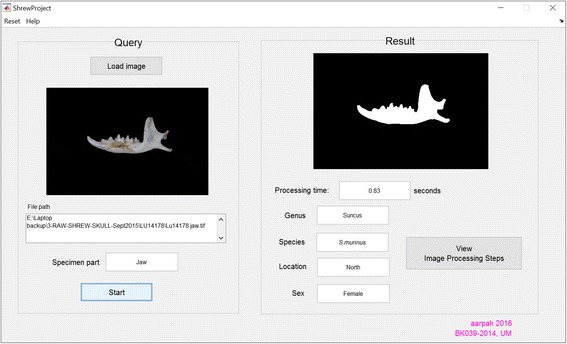
Species identification based on region and sex. Interface for user to query the system. User can upload an image of unknown specimen and the system will return the identified specimen with relevant information

**Fig. 9:**
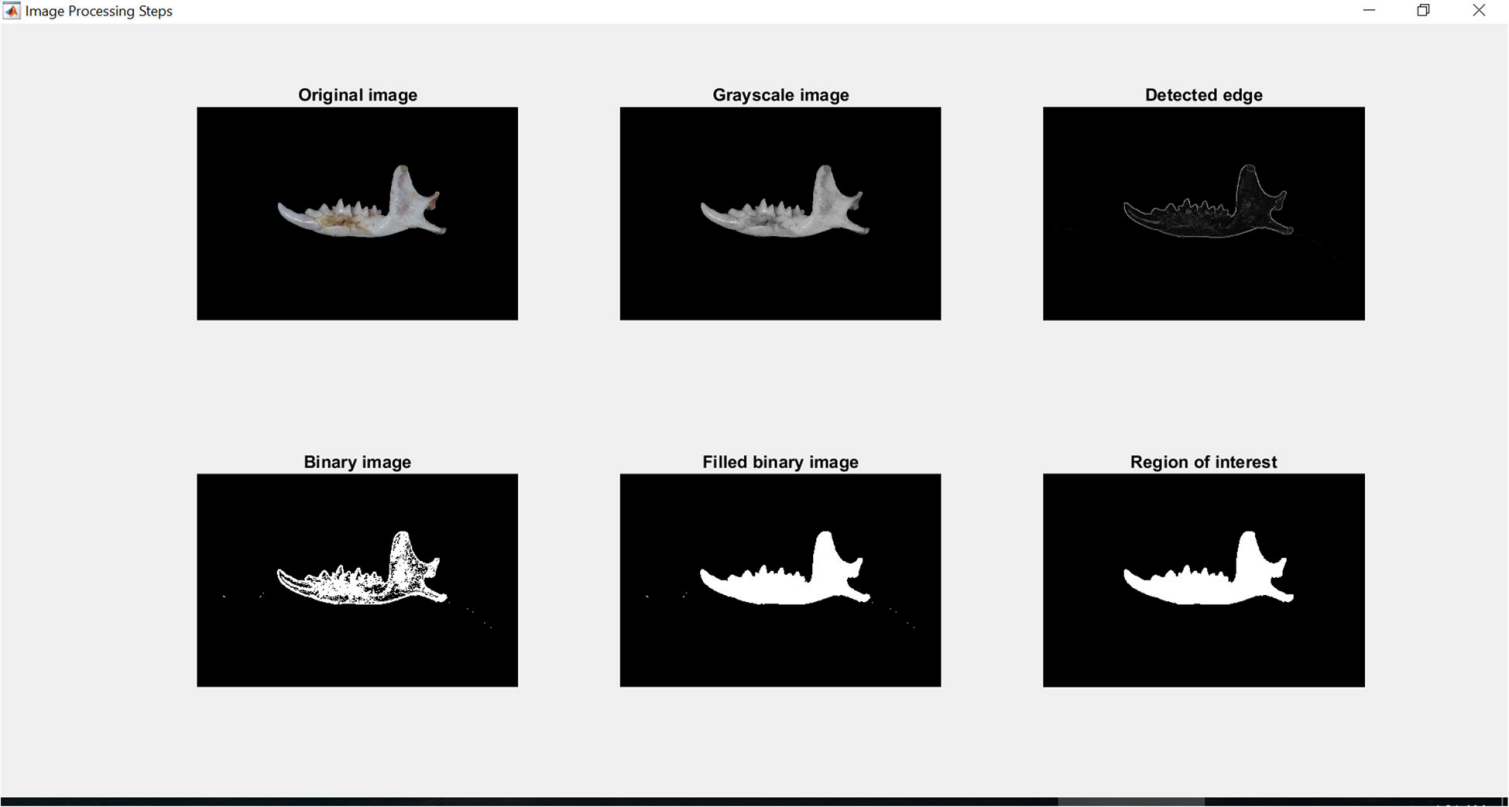
Result of image segmentation process. Interface to ensure that the correct query images are selected

As a proof-of-principle that this approach could be applied for the automated classification and identification using shape characteristics, a stand-alone identification system is developed as shown in Figs. 8 and 9. This system allows user to upload an image as unknown specimen, detect the view, extract features, and obtain the location and sex of identified specimen.

Graphical User Interface (GUI) was built to complete the identification system at the users’ side. The GUI enabled users to know the region and sex of a particular specimen with just a few clicks. The GUI is even suitable for someone with minimum biological knowledge as the system will first identify the query image before getting the right neural network to classify the correct region and sex. Not only the result images, users can also view the original query images and the result images in each of the image processing steps; this can ensure that the correct query images are selected and no mistake in image processing steps done by the system.

### The house shrew image database

**Fig. 10:**
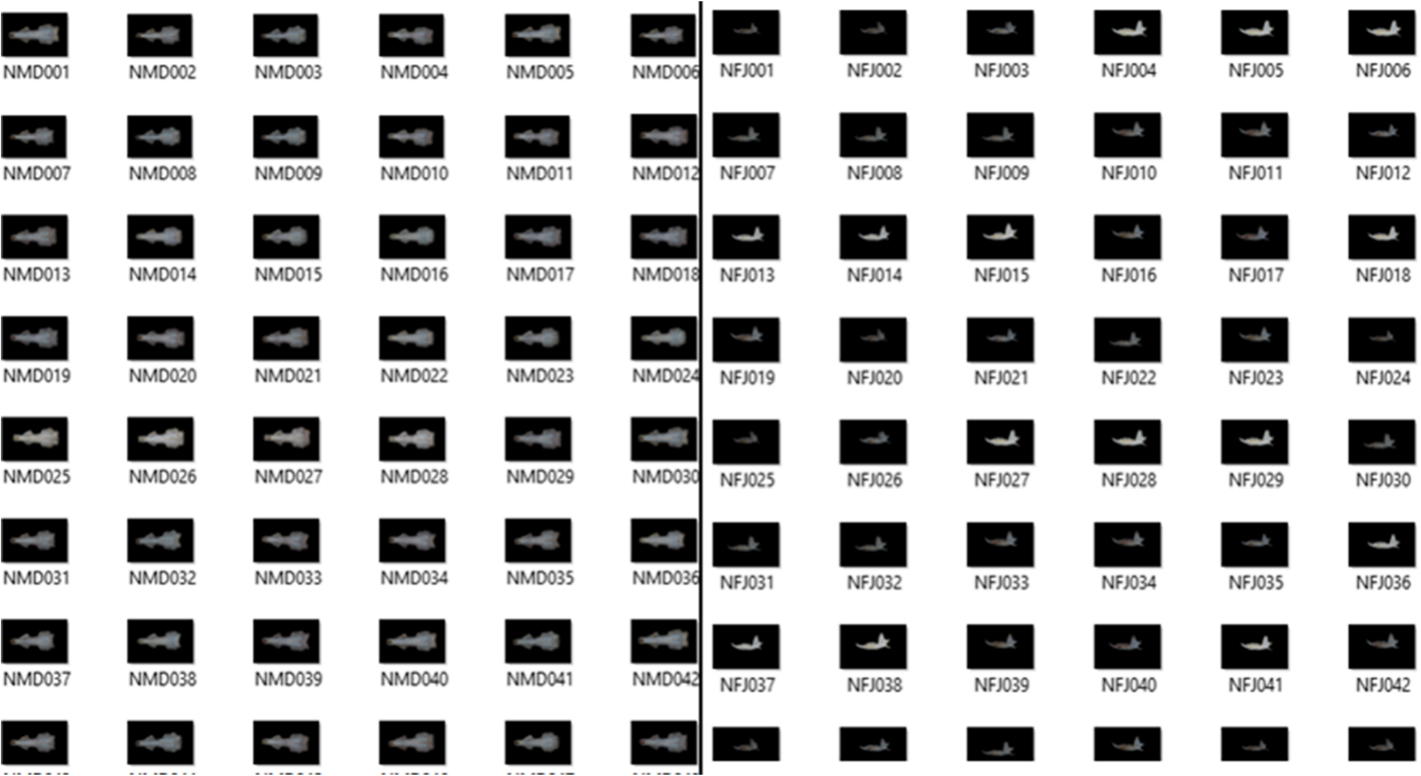
Images in the database. More than 1000 images were deposited in the image database, comprising of body and skull images of 200 specimens in various positions

The availability of biological specimens had meaningful support for this work. Thus, a comprehensive house shrew digital image database was constructed. Figure 10 shows some of images in the database. Currently more than 1000 images were deposited in the database, comprising of body and skull images of 200 specimens in various positions.

## Discussion

In this paper we report the classification of different populations of *S. murinus* using shape characteristics that were extracted from skull digital images and ANN. To ensure success, a set of features comprising of convex area, major axis length, minor axis length, perimeter, equivalent diameter and extent should be used. For both Training and Testing data sets, these features were extracted automatically and used to compose a 7D feature vector. The feature vectors of Training data sets (combination of all views – D, J & L; D – NM, NF, SM & SF; J – NM, NF, SM & SF; L – NM, NF, SM & SF) were then trained using ANN classifier and four networks were created. Identification of specimens was performed using Testing data set through two stages of ANNs.

The approach described here is simple and permits a reliable identification of the *S. murinus* species population. Even though features used in this study are simple as provided in MATLAB, they have significance in representing the views because the views can be discriminated morphologically. Considering this study was based on shape characteristics, the rates obtained for correct classification of four trained networks and overall accuracy of specimen identification are more than 70%, it can be considered as a good result. It shows that in terms of the shape, there are substantial differences between the specimens from southern and northern as well as the sexes of male and female specimens. The most substantial difference is on the lateral and jaw views with accuracy of 88.3% and 85%, respectively. Besides that, the current implementation of image segmentation is computationally efficient, permitting a rapid features extraction process.

Supervised ANN classifier used in this study is a common classifier that has been used in many studies specifically for automated taxon identification to species level, such as [33, 41–44]. However, in previous studies, identification was performed on one network only as compared to this study; the identification was performed on two stages of ANNs, the identification based on the view shapes and specimen discrimination based on the regions and sexes, respectively. Previously, only one network was created for classification in this study. However, it turned into a very complex trained network with 12 classes, computationally inefficient and the rate obtained for correct classification was as low as 66.7%. Hence, this study is extendable by using different machine learning approaches such as Support vector machine (SVM), decision tree, logistic regression, k-Nearest Neighbour (KNN) and others.

There is no study has been conducted on shrew species identification using digital image processing. Most of shrew researchers practice their works based on taxonomic and molecular systematics in achieving species status such as [45–48]. Thus, the most important finding in this study is a new invasive process for identification, whereby it could be used to assist data analysis that can greatly reduce the time and cost of species identification. Early taxonomists were classifying specimen populations based on morphological characters such as fur colour variations and body size [49, 50]. The other method to support this is through molecular works. However, these methods generally require complicated procedures since one has to perform data sampling from a broad range of geographical areas and to analyse a huge amount of information in identifying a species. This involves much effort, time, cost and manpower.

Few possible applications of this approach can be foreseen in a near future such as the image database. For each specimen, it consists of the body and skull images. The body image was captured during sampling at the field and the skull image was captured after skull extraction process in the laboratory. Thus, there is a possibility to look on body image for species identification using the same algorithm. This approach can be easily extended to discriminate other shrew species because the system uses generic algorithm. A preliminary study on two shrew species namely *Suncus malayanus* and *Crocidura monticola* had been done to identify species based on skull images. However, this work was performed on small sample size, thus further works need to be done. Lastly, this approach can be applied to differentiate small mammals such as rat (in [51], rat identification using colour feature) and vole using more general shape characteristics.

## Conclusions

This paper proposes shape characteristics to represent morphology of dorsal, lateral and jaw of *S. murinus* skull. This shape characteristic was applied for the differentiation of both sexes from northern and southern populations of *S. murinus* in Peninsular Malaysia using two stages of ANN classifier and the results revealed a good reliability of feature set. Lastly, an application was built and available for scientific community. This automated system demonstrates the practicability of using computer-assisted systems in providing interesting alternative approach for rapid and easy identification of unknown species.

## References

[1] Castelli V, Bergman LD (2002). Image Databases: Search and Retrieval of Digital Imagery.

[2] Holt B, Hartwick L (1994). Visual Image Retrieval for Applications in Art and Art History. Proc. SPIE Storage and Retrieval for Image and Video Databases.

[3] Benjamin BK. Shape Representation for Image Retrieval. In: Castelli V, Bergman LD, editors. Image Databases: Search and Retrieval of Digital Imagery. New York: John Wiley & Sons, Inc; 2002. p. 345–72.

[4] Erickson LA (2014). Incidental Findings in Medical Imaging and Genetic Testing: Opportunities and Challenges. Mayo Clin Proc.

[5] Orme NM, Wright TC, Harmon GE, Nkomo VT, Williamson EE, Sorajja P, Young PM (2014). Imaging Pandora's Box: Incidental Findings in Elderly Patients Evaluated for Transcatheter Aortic Valve Replacement. Mayo Clin Proc.

[6] Naidu SP, Margot JL, Taylor PA, Nolan MC, Busch MW, Benner LAM, Magri C (2015). Radar Imaging and Characterization of the Binary Near-Earth Asteroid (185851) 2000 DP107. Astron. J..

[7] Chen Z, Ma L, Xu L, Tan CM, Yan YH (2016). Imaging and representation learning of solar radio spectrums for classification. Multimedia Tools Appl.

[8] Arellano P, Tansey K, Balzter H, Boyd DS (2015). Detecting the effects of hydrocarbon pollution in the Amazon forest using hyperspectral satellite images. Environ Pollut.

[9] Qin Q, Zhang Z, Chen L, Wang N, Zhang C (2016). Oil and gas reservoir exploration based on hyperspectral remote sensing and super-low-frequency electromagnetic detection. J. Appl. Remote. Sens..

[10] Chaudhuri S, Middey A (2013). Nowcasting lightning flash rate and peak wind gusts associated with severe thunderstorms using remotely sensed TRMM-LIS data. Int J Remote Sensing.

[11] Liu JC, Liou YA, Wu MX, Lee YJ, Cheng CH, Kuei CP, Hong RM (2015). Analysis of Interactions Among Two Tropical Depressions and Typhoons Tembin and Bolaven (2012) in Pacific Ocean by Using Satellite Cloud Images. IEEE Trans. Geosci. Remote Sens..

[12] Craig AC (2002). The Implementation of Digital Photography in Law Enforcement and Government.

[13] Singh R (2005). Unconstrained face recognition for law enforcement applications.

[14] White DJ, Take WA, Bolton MD (2003). Soil deformation measurement using particle image velocimetry (PIV) and photogrammetry. Geotechnique.

[15] Chung SO, Cho KH, Cho JW, Jung KY, Yamakawa T (2012). Soil Texture Classification Algorithm Using RGB Characteristics of Soil Images. J Fac Agriculture Kyushu University.

[16] Ramapriyan HK, Castelli V, Bergman LD (2002). Satellite Imagery in Earth Science Applications. Image Databases: Search and Retrieval of Digital Imagery.

[17] Blanco-Gonzalo R, Poh N, Wong R, Sanchez-Reillo R (2015). Time evolution of face recognition in accessible scenarios. Human-Centric Comput Inf Sci.

[18] Ouarda W, Trichili H, Alimi AM, Solaiman B (2016). Towards A Novel Biometric System For Smart Riding Club. J Inf Assurance Security.

[19] Forsyth DA, Ponce J (2002). Computer Vision: A Modern Approach.

[20] Gonzalez RC, Woods RE (2010). Digital Image Processing.

[21] Yanhua Y, Chun C, Chun-Tak L, Hong F, Zheru C (2004). A computerized plant species recognition system. Proceedings of the International Symposium on Intelligent Multimedia, Video and Speech Processing.

[22] Moreno R, Grana M, Veganzones MA (2007). A Remote Mycological Assistant. 4th IEEE Workshop on Intelligent Data Acquisition and Advanced Computing Systems: Technology and Applications.

[23] Lang M, Kosch H, Stars S, Kettner C, Lachner J, Oborny D (2007). Recognition of Botanical Bloom Characteristics from Visual Features. Eighth International Workshop on Image Analysis for Multimedia Interactive Services.

[24] Kebapci H, Yanikoglu B, Unal G (2009). Plant image retrieval using color and texture features. 24th International Symposium on Computer and Information Sciences.

[25] Yang YS, Park DK, Kim HC, Choi MH, Chai JY (2001). Automatic identification of human helminth eggs on microscopic fecal specimens using digital image processing and an artificial neural network. IEEE Trans. Biomed. Eng..

[26] Wang J, Ji L, Liang A, Yuan D (2012). The identification of butterfly families using content-based image retrieval. Biosyst Eng.

[27] Sheikh AR, Lye MH, Mansor S, Fauzi MFA, Anuar FM (2011). A content based image retrieval system for marine life images. IEEE 15th International Symposium on Consumer Electronics.

[28] DAISY: A Practical Tool for Automated Species Identification. http://www.tumblingdice.co.uk/daisy. Accessed 26 July 2016.

[29] Weeks PJD, O'Neill MA, Gaston KJ, Gauld ID (1999). Automating insect identification: exploring the limitations of a prototype system. J. Appl. Entomol.

[30] Gauld ID, O'Neill MA, Gaston KJ (2000). Driving miss daisy: the performance of an automated insect identification system.

[31] Watson AT (2002). Automated identification of living macrolepidoptera using image analysis.

[32] Watson AT, O'Neill MA, Kitching IJ (2004). Automated identification of live moths (Macrolepidoptera) using digital automated identification System (DAISY). Syst. Biodivers..

[33] O'Neill MA, Macleod N (2007). DAISY: A Practical Computer-Based Tool for Semi-Automated Species Identification Automated Taxon Identification. Automated Taxon Identification in Systematics: Theory, Approaches and Applications.

[34] Mayo M, Watson AT (2007). Automatic species identification of live moths. Knowl.-Based Syst..

[35] Pajak M (2000). Identification of British Bombus and Megabombus using DAISY.

[36] SPIDAhome. http://research.amnh.org/iz/spida/common/index.htm. Accessed 26 July 2016.

[37] Do MT, Harp JM, Norris KC (1999). A test of a pattern recognition system for identification of spiders. Bull Entomol Res.

[38] Schröder S, Drescher W, Steinhage V, Kastenholz B (1995). An Automated Method for the Identification of Bee Species (Hymenoptera: Apoidea). International Symposium on Conserving Europe's Bees.

[39] Adam T (2008). Using geometric morphometrics and standard morphometry to discriminate three honeybee subspecies. Apidologie.

[40] Features - MATLAB. http://www.mathworks.com/products/matlab/features.html. Accessed 26 July 2016.

[41] Fedor P, Malenovsky I, Vanhara J, Sierka W, Havel J (2008). Thrips (Thysanoptera) identification using artificial neural networks. Bull Entomol Res.

[42] Favaro L, Briefer EF, McElligott AG (2014). Artificial Neural Network Approach for Revealing Individuality, Group Membership and Age Information in Goat Kid Contact Calls. Acta Acustica United with Acustica.

[43] Joutsijoki H, Meissner K, Gabbouj M, Kiranyaz S, Raitoharju J, Aumlrje J, Juhola M (2014). Evaluating the performance of artificial neural networks for the classification of freshwater benthic macroinvertebrates. Eco Inform.

[44] Leow LK, Chew LL, Chong VC, Dillon SK (2015). Automated identification of copepods using digital image processing and artificial neural network. BMC Bioinf.

[45] Meegaskumbura S, Schneider CJ (2008). A taxonomic evaluation of the shrew Suncus montanus (Soricidae: Crocidurinae) of Sri Lanka and India. Ceylon J Sci.

[46] Meegaskumbura S, Meegaskumbura M, Schneider CJ (2010). Systematic relationships and taxonomy of Suncus montanusand S. murinus from Sri Lanka. Mol Phylogenet Evol.

[47] Esselstyn JA, Maharadatunkamsi Achmadi AS, Siler CD, Evans BJ (2013). Carving out turf in a biodiversity hotspot: Multiple previously unrecognized shrew species co-occur on Java Island, Indonesia. Mol Ecol.

[48] Omar H, Hashim R, Bhassu S, Ruedi M (2013). Morphological and genetic relationships of the Crocidura monticola species complex (Soricidae: Crocidurinae) in Sundaland. Mamm. Biol..

[49] Medway L (1978). The Wild Mammals of Malaya and Singapore.

[50] Corbet GB, Hill JE (1992). Mammals of the Indomalayan Region: A Systematic Review.

[51] Falzon G, Meek PD, Vernes K, Bergman LD (2014). Computer-assisted identification of small Australian mammals in camera trap imagery.

